# Case Report: A giant ruptured splenic hydatic cyst in a patient with a complete situs inversus: Diagnostic challenge and intra-operative difficulties

**DOI:** 10.12688/f1000research.159480.1

**Published:** 2024-12-04

**Authors:** Amina Chaka, Wael Boujelbène, Amin Chaabouni, Mohamed Ali Bahloul, Nizar Kardoun, Salah Boujelben

**Affiliations:** 1 General Surgery, Department-Habib Bourguiba Hospital, Universite de Sfax Faculte de Medecine de Sfax, Sfax, Sfax, Tunisia

**Keywords:** spleen, hydatid cyst, echinococcosis, situs inversus, splenectomy, case report

## Abstract

The splenic localization of hydatid cysts is extremely rare.

A 50-year-old obese female who consults with a painful and febrile syndrome of the right hypochondrium. Abdominal ultrasound and a CT scan computed tomography revealed a complete situs inversus, a mass of the right hypochondrium measuring 152 mm with membrane detachment, and infiltration of the surrounding fat, evoking a type II complicated splenic hydatic cyst.

The patient was operated on in an emergency via midline laparotomy. Exploration revealed situs inversus, an angiant cyst of the spleen. Exposition of the splenic pedicle is difficult. The samples were then infected. Total splenectomy was performed. The postoperative period was unproblematic, and the patient was discharged with antibiotic and antiparasitic treatment and habitual vaccination.

## Introduction

Splenic hydatic localization is extremely rare, with a worldwide incidence rate of 0.5%-4%. ^1^ Abdominal left hypochondrium pain, mass, and fortuitous discoveries are the most frequently discovered complications ^1, 2^. However, right hypochondrium pain due to a splenic hydatic cyst associated with situs inversus is an exceptional finding. Here, we report the case of a 50-year-old female, who underwent surgery in our department for a complicated splenic hydatic cyst with situs inversus.

## Observation

### Patient information

A 50-year-old female, without no medical history presented to the emergency department with right hypochondrium pain.

### Clinical findings

On physical examination, the patient was febrile at 38,4°C; anicteric, with tenderness of the right hypochondrium on abdominal examination.

### Diagnostic assessment

Blood analysis showed a biological inflammatory syndrome. The liver test was normal.

In the face of a 50-year-old obese female who consulted for a painful and febrile syndrome of the right hypochondrium, an abdominal ultrasound was performed, which showed a complete situs inversus and a mass of the right hypochondrium with a membrane detachment, measuring 152 mm, evoking a type II splenic hydatic cyst.

**Figure 1.**
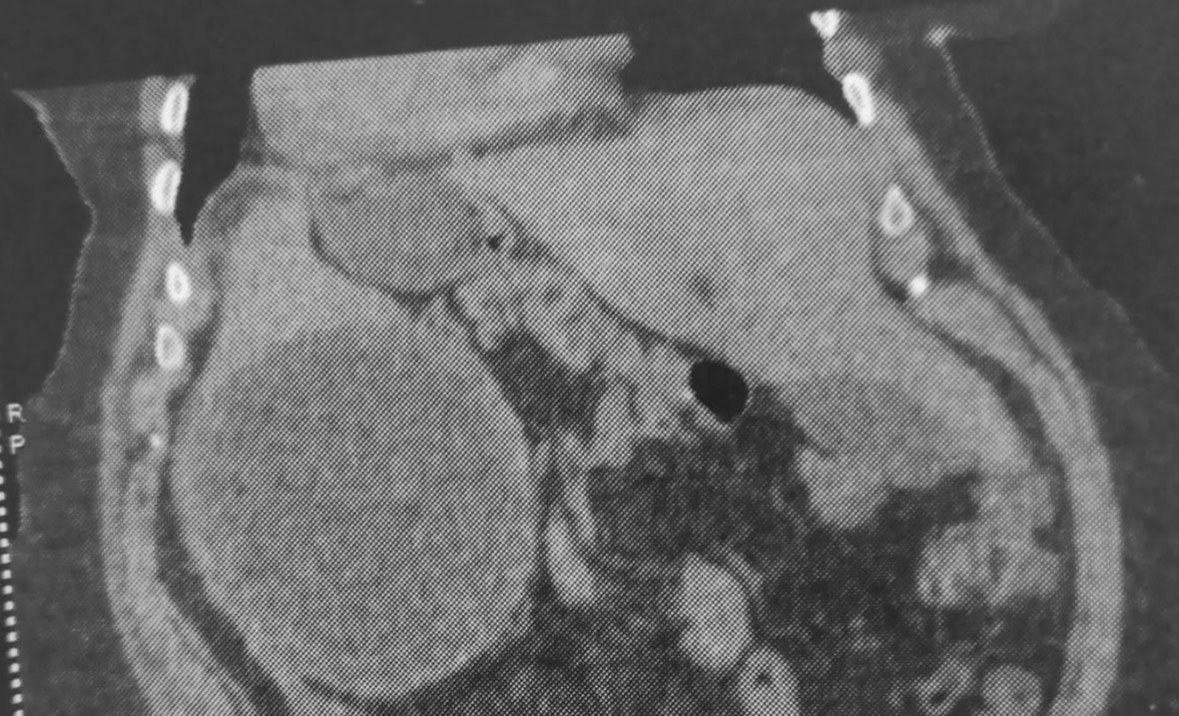
Splenic hydatid cyst cracking in a complete situs inversus 1.

**Figure 2.**
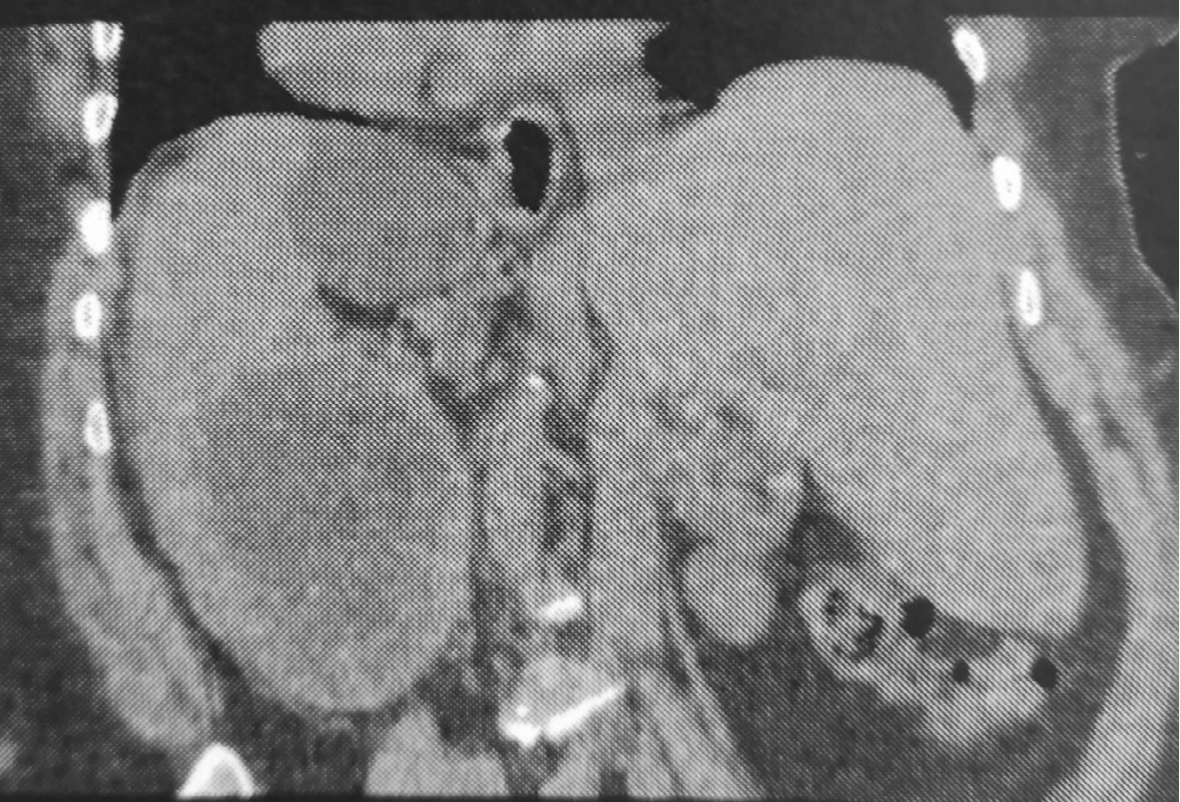
Splenic hydatid cyst cracking in a complete situs inversus 2.

Computed tomography (CT) revealed a splenic cystic formation, containing a membrane detachment, measuring 15 cm, evoking a type II splenic hydatic cyst with an infiltration of the surrounding fat, evoking a complication: hydatid cyst cracking ( Figure 1, 2).

### Therapeutic intervention

The patient underwent an emergency midline laparotomy. The exploration revealed a situs inversus, a voluminous splenic cyst occupying over 80% of the splenic volume. Exposition of the splenic pedicle is difficult. The cysto-parietal and cysto-visceral adherences, giant size of the cyst, and obesity prevented good exposure, which led to the decision to empty the cyst content after protecting the operating field with a field soaked in hypertonic serum. The samples were then infected.

Equally, the choice of the type of surgery, whether a total splenectomy or a protruding dome resection in an emergency context with complications such as cracking and surinfection, was not easy.

However, in the face of an emergency, the primary localization in the spleen, we performed a total splenectomy that allowed healing of the infested organ and avoided recurrence and surinfection of the residual cavity.

**Figure 3.**
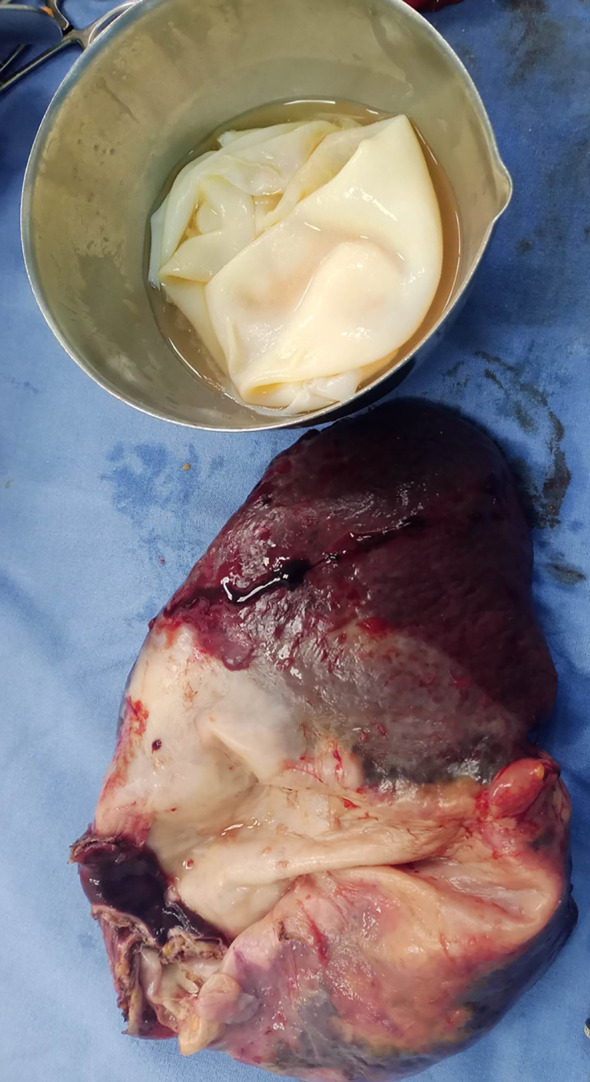
The splenectomy specimen and the proligere membrane of the hydatic cyst.

The overture of the cyst objectified the proligere membrane ( Figure 3).

### Follow up and outcomes

The post-operative period was unproblematic, and the patient was discharged with antibiotic and antiparasitic treatment and habitual vaccination.

## Discussion

Hydatic cysts are a common pathology in endemic countries. The most frequent locations are the liver and lungs. ^2^ Splenic localization is extremely rare, with a worldwide incidence rate of 0.5%-4%. ^1^

Based on the literature of some published cases of splenic primary localization, the pain, discovery of a left hypochondrium mass, and fortuitous discoveries are the most frequent discovery circumstances or during complications such as infection and splenic abscess, rupture with an anaphylactic shock, and dissemination to other organs. ^1, 2^

Ultrasound, computed tomography, and magnetic resonance imaging of the abdomen allow for diagnosis by objectifying membrane detachment and calcifications on the daughter vesicle wall. ^2, 3^

The treatment of splenic hydatic cysts is surgical. Total splenectomy has the advantage of avoiding recurrences. Protruuding dome resection has the advantage of being a conservative intervention of the organ and its functions and is slightly hemorrhagic at the cost of a considerable rate of residual cavity surinfection. ^4– 6^

The surgical approach depends on the localization of the splenic hydatic cyst(s) and its association with other cystic localizations. ^4, 7^ The laparoscopic approach is realizable in almost all cases, with good short-term and long-term results. ^6– 8^

To our knowledge, this is the first case of a giant splenic hydatic cyst and situs inversus.

## Conclusion

Isolated splenic hydatid cysts are uncommon and present significant challenges in both diagnosis and surgical intervention. Advanced imaging techniques, particularly computed tomography (CT), play a pivotal role in accurately identifying the condition and planning the appropriate treatment strategy. In this case, preoperative imaging not only confirmed the diagnosis but also provided valuable insights into the cyst’s size, location, and relationship with adjacent structures, which were critical for minimizing intraoperative risks and guiding the surgical approach.

## Patient perspective

The patient was satisfied with treatment with good follow-up after one year.

## Informed consent statement

Written informed consent for publication of their clinical details and clinical images was obtained from the patient.

## Data Availability

No data are associated with this article.
