# Case Report: A giant ruptured splenic hydatic cyst in a patient with a complete situs inversus: Diagnostic challenge and intra-operative difficulties

**DOI:** 10.12688/f1000research.159480.2

**Published:** 2025-01-10

**Authors:** Amina Chaka, Wael Boujelbène, Amin Chaabouni, Mohamed Ali Bahloul, Nizar Kardoun, Salah Boujelben

**Affiliations:** 1 General Surgery, Department-Habib Bourguiba Hospital, Universite de Sfax Faculte de Medecine de Sfax, Sfax, Sfax, Tunisia

**Keywords:** spleen, hydatid cyst, echinococcosis, situs inversus, splenectomy, case report

## Abstract

The splenic localization of hydatid cysts is extremely rare. A 50-year-old obese female who consults with a painful and febrile syndrome of the right hypochondrium. Abdominal ultrasound and a CT scan computed tomography revealed a complete situs inversus, a mass of the right hypochondrium measuring 152 mm with membrane detachment, and infiltration of the surrounding fat, evoking a type II complicated splenic hydatic cyst. The patient was operated on in an emergency via midline laparotomy. Exploration revealed situs inversus, an angiant cyst of the spleen. Exposition of the splenic pedicle is difficult. The samples were then infected. Total splenectomy was performed. The postoperative period was unproblematic, and the patient was discharged with antibiotic and antiparasitic treatment and habitual vaccination.

## Introduction

Splenic hydatic localization is extremely rare, with a worldwide incidence rate of 0.5%-4%. ^1^ Abdominal left hypochondrium pain, mass, and fortuitous discoveries are the most frequently discovered complications. ^1, 2^ However, right hypochondrium pain due to a splenic hydatic cyst associated with situs inversus is an exceptional finding. Here, we report the case of a 50-year-old female, who underwent surgery in our department for a complicated splenic hydatic cyst with situs inversus.

## Observation

### Patient information

A 50-year-old female, without no medical history presented to the emergency department with right hypochondrium pain.

### Clinical findings

On physical examination, the patient was febrile at 38,4°C; anicteric, with tenderness of the right hypochondrium on abdominal examination. The hemodynamic status was stable.

### Diagnostic assessment

Blood analysis showed a biological inflammatory syndrome. The liver test was normal.

In the face of a 50-year-old obese female who consulted for a painful and febrile syndrome of the right hypochondrium, an abdominal ultrasound was performed, which showed a complete situs inversus and a mass of the right hypochondrium with a membrane detachment, measuring 152 mm, evoking a type II splenic hydatic cyst.

**Figure 1.**
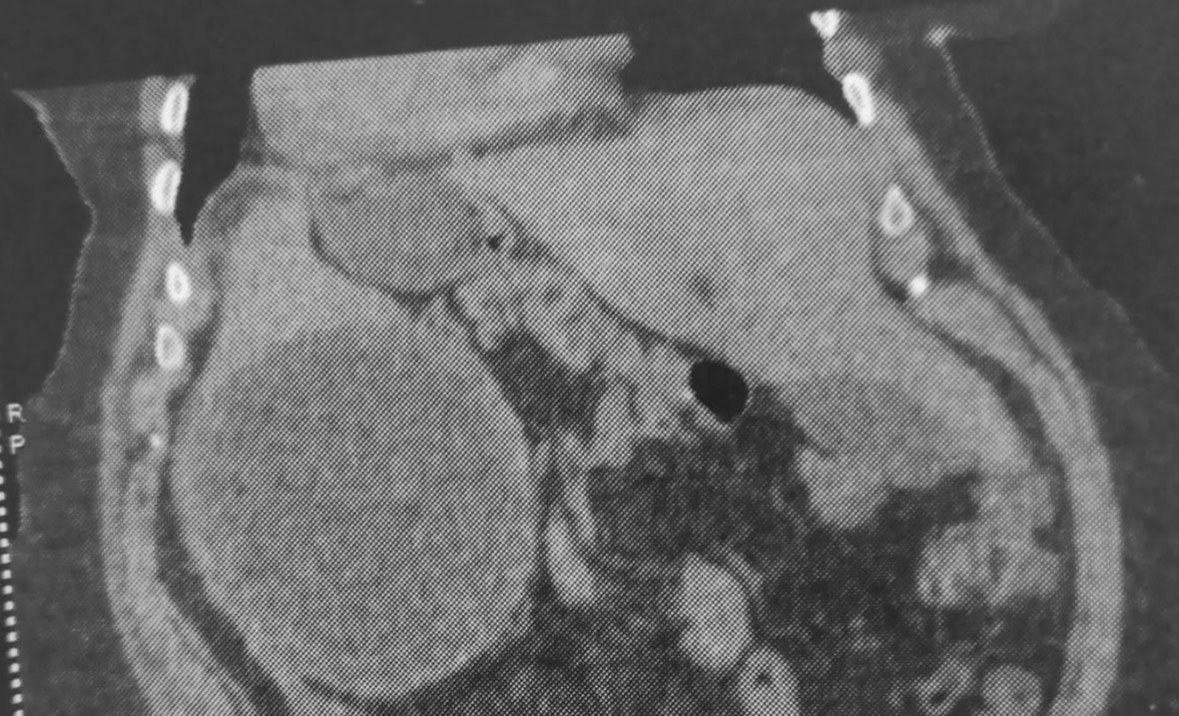
Splenic hydatid cyst cracking in a complete situs inversus 1.

**Figure 2.**
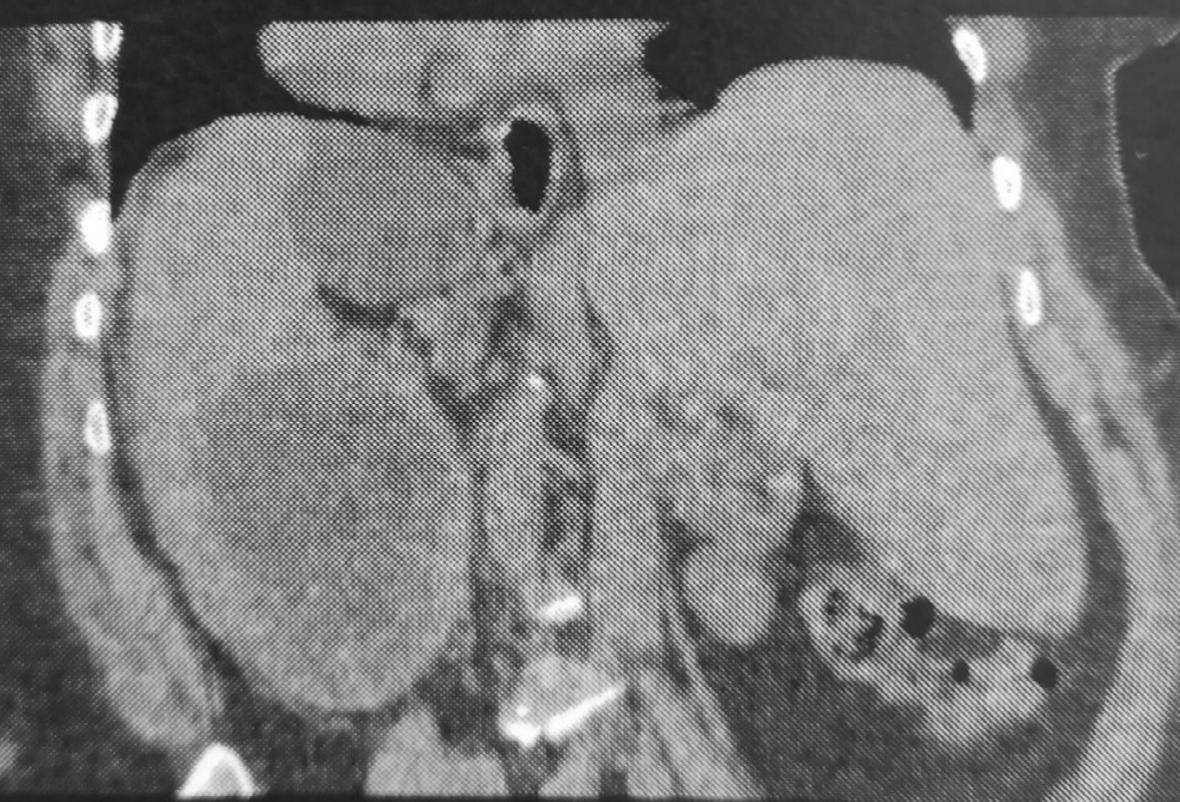
Splenic hydatid cyst cracking in a complete situs inversus 2.

Computed tomography (CT) revealed a large cystic formation in the spleen, measuring 15 cm in its largest dimension. The cyst exhibited a detached internal membrane, a characteristic sign suggestive of a type II hydatid cyst according to the Gharbi classification. Additionally, there was evidence of surrounding fat stranding and infiltration, indicative of local inflammatory changes. These findings strongly suggest a complication, specifically a hydatid cyst cracking ( Figures 1, 2).

### Therapeutic intervention

The patient underwent an emergency midline laparotomy. The exploration revealed a situs inversus, a voluminous splenic cyst occupying over 80% of the splenic volume. Exposition of the splenic pedicle is difficult. The cysto-parietal and cysto-visceral adherences, giant size of the cyst, and obesity prevented good exposure, which led to the decision to empty the cyst content after protecting the operating field with a field soaked in hypertonic serum. The samples were then infected.

Equally, the choice of the type of surgery, whether a total splenectomy or a protruding dome resection in an emergency context with complications such as cracking and surinfection, was not easy.

However, in the face of an emergency, the primary localization in the spleen, we performed a total splenectomy that allowed healing of the infested organ and avoided recurrence and surinfection of the residual cavity.

**Figure 3.**
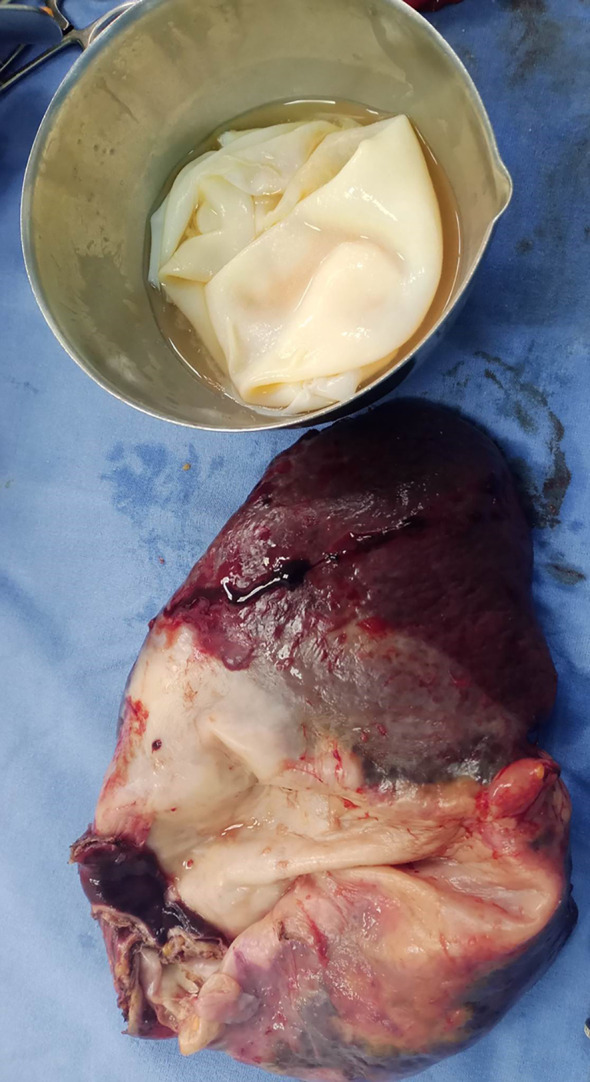
The splenectomy specimen and the proligere membrane of the hydatic cyst.

The overture of the cyst objectified the proligere membrane ( Figure 3).

### Follow up and outcomes

The post-operative period was unproblematic, and the patient was discharged with antibiotic and antiparasitic treatment and habitual vaccination.

Treatment with albendazole 400 mg twice daily was initiated from post-operative day 1, with cycles consisting of three 28-day treatments at 2-week intervals. Serum anti-Echinococcus antibody titers (hemagglutination test and ELISA) were positive in the immediate postoperative period (titers of 1/1280 and 1.9, respectively). Subsequently, the titers gradually decreased until achieving a negative serology at 2 years post-operatively. Liver function tests and complete blood counts were performed after each cycle and did not reveal any abnormalities during the treatment period. The abdominal CT scan did not reveal any recurrence of cystic echinococcosis in the thoraco-abdominal regions, particularly in the peritoneal area, with a current follow-up period of 3 years.

## Discussion

Hydatic cysts are a common pathology in endemic countries. The most frequent locations are the liver and lungs. ^2^ Splenic localization is extremely rare, with a worldwide incidence rate of 0.5%-4%. ^1^

To our knowledge, this is the first reported case of a giant splenic hydatid cyst associated with situs inversus. In our case, the cyst was also ruptured into the abdomen, which posed both a diagnostic and therapeutic challenge.

Based on the literature of some published cases of splenic primary localization, the pain, discovery of a left hypochondrium mass, and fortuitous discoveries are the most frequent discovery circumstances or during complications such as infection and splenic abscess, rupture with an anaphylactic shock, and dissemination to other organs. ^1, 2^

Ultrasound, computed tomography, and magnetic resonance imaging of the abdomen allow for diagnosis by objectifying membrane detachment and calcifications on the daughter vesicle wall. ^2, 3^ In the case of a complicated cyst, cross-sectional imaging, particularly CT scans, can establish the diagnosis in an emergency while also allowing for the assessment of the cyst’s location and its anatomical relationships. ^2, 3^

The treatment of splenic hydatic cysts is surgical. Total splenectomy has the advantage of avoiding recurrences. Protruuding dome resection has the advantage of being a conservative intervention of the organ and its functions and is slightly hemorrhagic at the cost of a considerable rate of residual cavity surinfection. ^4– 6^

The surgical approach depends on the localization of the splenic hydatic cyst(s) and its association with other cystic localizations. ^4, 7^ The laparoscopic approach is realizable in almost all cases, with good short-term and long-term results. ^6– 8^

Regarding complicated cysts, the treatment of a ruptured hydatid cyst typically relies on urgent surgical intervention, ^4– 6^ followed by medical therapy to prevent and manage peritoneal echinococcosis. ^9^ However, Carola Buscemi et al. ^9^ reported a case where prolonged treatment with albendazole was employed over 10 years for peritoneal, hepatic, and splenic hydatidosis, including a ruptured cyst. The albendazole protocol consisted of 400 mg administered twice daily for three cycles of 28 days each, with a 14-day break between cycles. This treatment was well-tolerated, and the hydatidosis showed favorable progression under this regimen.

Such cases highlight the potential for medical therapy as a complementary or alternative approach in selected cases, particularly when surgery poses high risks or is incomplete. However, further randomized prospective studies are needed to establish standardized protocols and optimize the management of this common yet complex condition.

## Conclusion

Isolated splenic hydatid cysts are uncommon and present significant challenges in both diagnosis and surgical intervention. Advanced imaging techniques, particularly computed tomography (CT), play a pivotal role in accurately identifying the condition and planning the appropriate treatment strategy. In this case, preoperative imaging not only confirmed the diagnosis but also provided valuable insights into the cyst’s size, location, and relationship with adjacent structures, which were critical for minimizing intraoperative risks and guiding the surgical approach.

## Patient perspective

The patient was satisfied with treatment with good follow-up after one year.

## Informed consent statement

Written informed consent for publication of their clinical details and clinical images was obtained from the patient.

## Data Availability

No data are associated with this article.
