# Supplementary material for: Case Report: A giant ruptured splenic hydatic cyst in a patient with a complete situs inversus: Diagnostic challenge and intra-operative difficulties
Source: F1000Res. 2025 Jun 2;13:1480. Originally published 2024 Dec 4. [Version 3] doi: 10.12688/f1000research.159480.3 (PMC11934099; doi:10.12688/f1000research.159480.3)
Supplement: Peer review history

# Reviewer report: Reviewer response for version 3

**DOI:** 10.5256/f1000research.183133.r389324

**Published:** 2025-06-06

**Authors:** Ali Bilal Ulas

**Affiliations:** 1 Ataturk University, Erzurum, Turkey

Dear Editor and Authors,

 I thank you for addressing my suggestions thoroughly in the revised version of the manuscript. The updated text shows clear improvements in structure, clarity, and scientific rigor. The discussion is now better organized, the language has been polished, and the significance of the case particularly in relation to hydatid cysts in atypical locations and surgical management in situs inversus is well articulated.

 Best regards,

Are enough details provided of any physical examination and diagnostic tests, treatment given and outcomes?

Yes

Is the case presented with sufficient detail to be useful for other practitioners?

Yes

Is sufficient discussion included of the importance of the findings and their relevance to future understanding of disease processes, diagnosis or treatment?

Yes

Is the background of the case’s history and progression described in sufficient detail?

Partly

Reviewer Expertise:

Thoracic Surgery

I confirm that I have read this submission and believe that I have an appropriate level of expertise to confirm that it is of an acceptable scientific standard.

# Reviewer report: Reviewer response for version 2

**DOI:** 10.5256/f1000research.176728.r371891

**Published:** 2025-03-22

**Authors:** Ali Bilal Ulas

**Affiliations:** 1 Ataturk University, Erzurum, Turkey

Thanks for this manuscript that presents a case report of a giant ruptured splenic hydatid cyst in a patient with complete situs inversus, which is a rare and clinically significant condition.

 The case is highly unusual due to the combination of a giant splenic hydatid cyst and complete situs inversus, making it a valuable contribution to the medical literature. The report provides a thorough account of the diagnostic process, including imaging findings, and the surgical approach, which is beneficial for practitioners facing similar challenges.

 The introduction could benefit from a more focused thesis statement that explicitly outlines the key aspects of the case report such as the diagnostic challenges, surgical difficulties, and the uniqueness of the presentation.

 The flow of information is sometimes disjointed. For instance, the discussion section could be better organized to present a more logical argument.

 How does this case contribute to the understanding of hydatid cysts in atypical locations? What are the specific challenges posed by situs inversus in surgical interventions, and how can they be mitigated in future cases?

 The manuscript suffers from numerous grammatical errors, typos, and awkward phrasing.  For example:

 "A 50-year-old female, without no medical history presented to the emergency department with right hypochondrium pain." (Double negative: "without no" should be "with no").

 "The samples were then infected." (Unclear phrasing: It should be clarified whether the samples were found to be infected or if they became infected during the procedure).

 "The cyst exhibited a detached internal membrane, a characteristic sign suggestive of a type III hydatid cyst according to the Gharbi classification." (This sentence is somewhat redundant and could be streamlined for clarity).

Are enough details provided of any physical examination and diagnostic tests, treatment given and outcomes?

Yes

Is the case presented with sufficient detail to be useful for other practitioners?

Yes

Is sufficient discussion included of the importance of the findings and their relevance to future understanding of disease processes, diagnosis or treatment?

Yes

Is the background of the case’s history and progression described in sufficient detail?

Partly

Reviewer Expertise:

Thoracic Surgery

I confirm that I have read this submission and believe that I have an appropriate level of expertise to confirm that it is of an acceptable scientific standard, however I have significant reservations, as outlined above.

# Reviewer report: Reviewer response for version 1

**DOI:** 10.5256/f1000research.175212.r349374

**Published:** 2025-01-07

**Authors:** Silvio Buscemi

**Affiliations:** 1 AOU Policlinico "P. Giaccone", Policlinico University Hospital, University of Palermo, Palermo, Italy

The case described is very interesting and well-written. I have some general considerations for you below.

 It is appropriate to discuss cystic echinococcosis in female with obesity.

 Given the unique nature of this case with situs inversus, including descriptive arrows in the CT images is essential and reassuring. This will provide clear visual guidance for the reader, enhancing their confidence in the case report.

 Please elaborate on the antiparasitic treatment used, including the specific regimen followed (it is important to continue the treatment after the cyst spontaneously ruptures to avoid possible dissemination).

 It is essential to document the changes in antibody titers and blood chemistry tests following surgical treatment and therapy (it would be appropriate to document how in the article, that could also be mentioned: Ref 1). This will not only inform the reader but also enhance their knowledge about the progression of the disease.

Are enough details provided of any physical examination and diagnostic tests, treatment given and outcomes?

Partly

Is the case presented with sufficient detail to be useful for other practitioners?

Partly

Is sufficient discussion included of the importance of the findings and their relevance to future understanding of disease processes, diagnosis or treatment?

Partly

Is the background of the case’s history and progression described in sufficient detail?

Yes

Reviewer Expertise:

internal medicine, obesity, metabolic disease

I confirm that I have read this submission and believe that I have an appropriate level of expertise to confirm that it is of an acceptable scientific standard.

## References

[1] : Very Prolonged Treatment with Albendazole of a Case of Disseminated Abdominal Cystic Echinococcosis. *Tropical Medicine and Infectious Disease* .2023;8(9) : 10.3390/tropicalmed8090449 10.3390/tropicalmed8090449 PMC1053463337755910

# Response

**Published:** 2025-01-07

**Authors:** Wael Boujelbène

**Affiliations:** general surgery, University of Sfax Faculty of Medecine of Sfax, Sfax, Sfax, Tunisia

Dear Dr. Silvio Buscemi,

 Thank you for your insightful comments and positive feedback regarding our case report. We appreciate your valuable suggestions, which have significantly improved the quality of our work. Please find our responses to your considerations below:

**1. Discussion of cystic echinococcosis in females with obesity**

 In the surgical treatment of abdominal cystic echinococcosis, obesity can complicate operative exposure due to increased intra-abdominal fat, making visualization and access to the surgical field more challenging. This raises the risk of spillage or injury to adjacent structures and may prolong operative time. Obesity-related comorbidities can further increase perioperative risks, highlighting the need for careful preoperative planning and surgical expertise.

 2. **Inclusion of descriptive arrows in CT images**

 We acknowledge the importance of providing clear visual guidance in the CT images. Descriptive arrows have been added to highlight key findings, especially given the unique presentation of situs inversus.

 3. **Details of the antiparasitic treatment regimen**

 We have expanded the description of the antiparasitic treatment regimen, specifying the protocol followed, including dosages, treatment duration, and intervals. This addition highlights the importance of continuing medical therapy after cyst rupture to prevent dissemination and recurrence.

 4. **Documentation of antibody titers and blood chemistry changes**

 We have included detailed data on the evolution of antibody titers and blood chemistry tests post-surgery and during therapy. These additions are presented in  the results sections to provide a comprehensive view of disease progression and response to treatment.

 5. **References**

 We have incorporated your suggested reference to complement our discussion and to underline the importance of prolonged treatment with albendazole in managing disseminated cystic echinococcosis.

 6. **Additional improvements**

 We have ensured that the background, physical examination findings, diagnostic tests, and treatment outcomes are presented in greater detail. The discussion has also been expanded to highlight the importance of our findings for future understanding and management of similar cases.We hope these revisions address your comments and enhance the quality and utility of our case report for other practitioners. Thank you for your time, expertise, and valuable feedback.

 Sincerely,

# Reviewer report: Reviewer response for version 1

**DOI:** 10.5256/f1000research.175212.r349375

**Published:** 2024-12-28

**Authors:** Selmy Awad

**Affiliations:** 1 Mansoura University Hospitals, El-Gomhouria Street, El-Mansoura, Egypt

Thanks for the novel case as an incidence and location.

 many typos and grammar mistakes are abundant.

 What is the role of medical treatment in preoperative preparation and post-operative regimens?

 please follow the standards for writing case reports

Are enough details provided of any physical examination and diagnostic tests, treatment given and outcomes?

Yes

Is the case presented with sufficient detail to be useful for other practitioners?

Partly

Is sufficient discussion included of the importance of the findings and their relevance to future understanding of disease processes, diagnosis or treatment?

No

Is the background of the case’s history and progression described in sufficient detail?

Yes

Reviewer Expertise:

acute care ,trauma ,laparoscopy

I confirm that I have read this submission and believe that I have an appropriate level of expertise to confirm that it is of an acceptable scientific standard, however I have significant reservations, as outlined above.

# Response

**Published:** 2025-01-07

**Authors:** Wael Boujelbène

**Affiliations:** general surgery, University of Sfax Faculty of Medecine of Sfax, Sfax, Sfax, Tunisia

Dear Dr. Selmy Awad:

 Thank you for your valuable feedback on the case report. I appreciate your thoughtful insights and constructive criticism.

 1. Typos and Grammar Mistakes: We acknowledge that there were some typos and grammatical errors in the manuscript. These will be carefully reviewed and corrected to ensure clarity and professionalism in the final version.

 2. Role of Medical Treatment in Preoperative and Postoperative Regimens: You have raised an important point regarding the role of medical treatment in both preoperative preparation and postoperative regimens. We will include a more comprehensive discussion on the medical management, emphasizing its significance in patient preparation before surgery, as well as in the postoperative care to enhance recovery and prevent complications.

 3. Standards for Writing Case Reports: We will ensure that the case report is fully aligned with the standards for writing case reports. This will involve revising the structure and adding any missing sections to enhance the clarity and scientific rigor.

 4. Importance of Findings and Relevance to Disease Understanding: We appreciate your observation about the lack of a detailed discussion regarding the importance of the findings and their relevance to future disease understanding. We will expand this section to explain the significance of the case in relation to broader disease processes, diagnosis, and treatment. This will help contextualize the findings for future research and clinical practice.

 5. Sufficiency of Details for Other Practitioners: We will review the report to ensure that enough practical insights and relevant details are provided for it to be a useful reference for others in the field.

 Thank you again for your review. We will address these concerns thoroughly in the revised version of the manuscript.
